# Development of the Hair & Scalp CARE questionnaire: Measuring the impact of hair and scalp issues on psychological wellbeing in healthy populations

**DOI:** 10.1111/ics.13070

**Published:** 2025-04-22

**Authors:** Alice Newton‐Fenner, William M. Hirst, Therese Jones, Margaret Scott, Carl Roberts, Monique A. M. Smeets, Jeremy Shen, Anna Thomas, Timo Giesbrecht

**Affiliations:** ^1^ Unilever Research & Development Port Sunlight UK; ^2^ Department of Psychological Sciences, Institute of Population Health University of Liverpool Liverpool UK; ^3^ Faculty of Social and Behavioural Sciences Utrecht University Utrecht the Netherlands; ^4^ Unilever Research & Development Trumbull Connecticut USA

**Keywords:** haircare, questionnaire validation, scalpcare, wellbeing

## Abstract

**Objective:**

Haircare cosmetic products are commonly reported to have a positive impact on psychological wellbeing. These effects are attributed to increased feelings of confidence and improved self‐esteem facilitated by improved hair and scalp condition. However, the causal relationship between hair and scalp health and psychological wellbeing is under‐researched. This paper reports the results of an extensive survey of haircare consumers in diverse populations using an exploratory Hair & Scalp CARE (Condition and Affective Response Evaluations) questionnaire.

**Method:**

Participants (*N* = 1184) completed an online 23‐item questionnaire designed to capture hair and scalp‐related wellbeing as an initial exploratory validation of Hair & Scalp CARE. For the analysis, the data were randomly split into 2 equal samples; Sample 1 provided the data for the initial exploratory factor analysis, and Sample 2 was used for confirmatory factor analysis. Participants also provided demographic information and completed the Sleep Health Index (SHI) and Perceived Stress Scale (PSS) to investigate sleep health and perceived stress.

**Results:**

Factor analysis provided a one‐factor solution, explaining 55% of the variance. The final version of the Hair & Scalp CARE questionnaire consisted of 21 items. The one‐factor structure was supported by confirmatory factor analysis. Correlational analyses demonstrated that higher scores on Hair & Scalp CARE were also associated with lower PSS scores and higher SHI scores.

**Conclusion:**

The Hair & Scalp CARE questionnaire is a valid tool for the assessment of the impact of hair and scalp condition on psychological wellbeing. The present data also suggest a relationship between hair and scalp wellbeing and other psychological wellbeing indicators, as healthier hair and scalp was also linked to lower levels of perceived stress and good sleep health. Hair & Scalp CARE could be used within a variety of further research designs to demonstrate the positive impact of cosmetic haircare products on wellbeing.

## Graphical abstract

The Hair & Scalp CARE questionnaire is a validated tool for assessing the impact of hair and scalp condition on psychological wellbeing. The present data also suggest greater hair and scalp wellbeing is linked to lower levels of perceived stress and good sleep health.

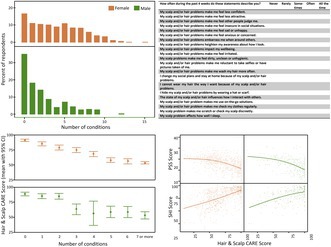

## Résumé

**Objectif:**

Les produits cosmétiques de soins capillaires sont généralement considérés comme ayant un impact positif sur le bien‐être psychologique. Ces effets sont attribués à une plus grande confiance en soi et à une meilleure estime de soi, favorisées par l'amélioration de l'aspect des cheveux et du cuir chevelu. Cependant, la relation de cause à effet entre des cheveux et un cuir chevelu sains et le bien‐être psychologique fait l'objet de peu de recherches. Cet article présente les résultats d'une enquête à grande échelle menée auprès de consommateurs de produits capillaires issus de diverses populations, à l'aide d'un questionnaire exploratoire Hair & Scalp CARE (*Condition and Affective Response Evaluations* [évaluations des problèmes et de la réponse affective]).

**Méthode:**

Les participants (*N* = 1184) ont répondu à un questionnaire en ligne à 23 items conçu pour mesurer le bien‐être lié aux cheveux et au cuir chevelu, dans le cadre d'une première validation exploratoire du questionnaire Hair & Scalp CARE. Pour l'analyse, les données ont été réparties de manière aléatoire en 2 échantillons égaux; l'échantillon 1 a fourni les données pour l'analyse factorielle exploratoire initiale et l'échantillon 2 a été utilisé pour l'analyse factorielle confirmatoire. Les participants ont également fourni des données démographiques et ont répondu à l'indice de l'état du sommeil (*Sleep Health Index*, SHI) et à l'échelle de stress perçu (*Perceived Stress Scale*, PSS) afin d'étudier l'état du sommeil et le stress perçu.

**Résultats:**

L'analyse factorielle a fourni une solution à un facteur, expliquant 55% de la variance. La version finale du questionnaire Hair & Scalp CARE comprenait 21 items. La structure à un facteur a été confirmée par l'analyse factorielle confirmatoire. Les analyses corrélatives ont montré que des scores plus élevés au questionnaire Hair & Scalp CARE étaient également associés à des scores plus faibles à l'échelle de stress perçu et à des scores plus élevés à l'indice de l'état du sommeil.

**Conclusion:**

Le questionnaire Hair & Scalp CARE est. un outil valable pour évaluer l'impact de l'état des cheveux et du cuir chevelu sur le bien‐être psychologique. Ces données laissent également penser à une relation entre le bien‐être en lien avec les cheveux et le cuir chevelu et d'autres indicateurs de bien‐être psychologique, des cheveux et un cuir chevelu plus sains étant également liés à des niveaux de stress perçus plus faibles et à un bon état du sommeil. Le questionnaire Hair & Scalp CARE pourrait être utilisé dans le cadre de diverses recherches ultérieures pour démontrer l'impact positif des produits cosmétiques capillaires sur le bien‐être.

## INTRODUCTION

Hair can be an important symbol of cultural identity, social status and expression of individuality [1]. It plays a vital role in age and gender identification [2, 3, 4, 5] and contributes to perceptions of overall health. For instance, long, shiny, thick and voluminous hair is often seen as a sign of physical wellbeing, a robust immune system and high fertility, which can enhance attractiveness as a potential mate [6, 7]. These perceptions are particularly strong among adolescents and young adults, as scalp hair is a crucial element of body image and sexual appeal [8, 9]. As a result, detrimental changes to one's hair and scalp, such as damage, age‐related thinning and dermatological conditions, can have significant psychological impacts on both men and women. For example, ‘bad hair days’ have been shown to have robust negative effects on self‐esteem, social insecurity and self‐criticism [10], with the impact of hair on self‐esteem being mediated by an individual's menstrual cycle [11]. Men typically attribute bad hair days to unruly, out‐of‐control and wild hair, while women associate them with frizzy, damaged and dull hair. Linked to this, hair appearance can be used as a social signal of health to inform judgements from others: Visible age‐related hair changes may be perceived as indicative of poor self‐care, with individuals with greying and thinning hair being rated as less attractive and older than they actually are [2, 12, 13].

In addition to poor hair quality, increased hair loss is also associated with lower self‐confidence, self‐consciousness, quality of life (QoL) and greater symptoms of anxiety and depression [14]. Previous studies report that men with male pattern baldness can experience up to a 75% decrease in confidence, particularly when interacting with the opposite gender, and younger men feel a more acute impact of hair loss on their self‐esteem [15, 16, 17]. Androgenetic alopecia, also known as female pattern hair loss, has significant negative impacts on psychological wellbeing in women, with one investigation observing 88% of the sample reporting adverse effects on daily living, 75% reporting lowered self‐esteem and 33% reporting an overall disturbance in QoL [18]. In clinical groups, individuals with lichen planopilaris (LPP), a form of scarring alopecia, also report higher scores of depression, anxiety and sleep complaints compared with matched controls [19]. Finally, a recent study comparing individuals with LPP to healthy controls found that lower self‐esteem scores, higher anxiety levels and higher scores in LPP activity were predictive of poorer QoL [20].

Scalp health is also vital, as dandruff will affect over 50% of the global adult population at some point in their lifetime [21]. The occurrence (and repeated reoccurrence) of dandruff, and its more severe manifestation seborrheic dermatitis, can lead to self‐consciousness and embarrassment. The associated increase of itching and scalp agitation can cause embarrassment in public [22, 23]. In one study, men with dandruff were perceived as less confident and exhibiting lower self‐esteem than men without dandruff, even when there were no self‐rated differences in confidence or self‐esteem between the two groups [24].

Good haircare practices can significantly increase self‐confidence: from routine self‐care rituals to dramatic, transformative haircuts signifying the start of a new life chapter. Consumers may also purchase cosmetic products and seek aesthetic treatments to enhance hair colour, thickness or shine with the aim to improve their self‐esteem. For example, the use of hair colouring products has been associated with improved QoL and wellbeing in female users [25, 26, 27]. According to the American Hair Loss Association, approximately $3.5 billion is spent on hair loss prevention in the United States annually [28]. Additionally, the World Health Organization (WHO) estimates that approximately 70% of adults have scalp and hair problems globally [29], emphasizing the widespread implications of any impact on psychological wellbeing. Therefore, there is a clear need for scientifically validated metrics that can quantify the impact of hair and scalp health on psychological wellbeing.

Much of the research to date has focused on the relationship between scalp and/or hair health and QoL within clinical samples to better understand the psychological impact of various dermatological conditions. The Dermatology Life Quality Index (DLQI) [30, 31], the Scalpdex [32] and the Skindex [33] are three of the most widely used self‐reported health‐related QoL instruments specific to dermatology. The DLQI assesses QoL across six domains: symptoms and feelings, treatment, daily activities, leisure, work/school and personal relationships. The Scalpdex measures three domains (symptoms, functioning and emotions), while the Skindex addresses eight domains: cognitive effects, social effects, depression, fear, embarrassment, anger, physical discomfort and physical limitations. Although these instruments are well validated and translated into various languages, the DLQI has been criticized for its failure to address the emotional and mental impact of the disease [34]. In comparison, the Skindex questionnaire family (e.g. Skindex‐29, Skindex‐17, Skindex‐16, Skindex‐mini) [33] is reported to better capture the emotional and mental health impact of dermatological conditions [35, 36, 37]. However, these instruments primarily focus on disease symptoms, functional impairments and treatment, and therefore may not be suitable for investigating healthy samples.

Conversely, generic QoL instruments fail to capture the extent and nuance of scalp and hair health‐related concerns on wellbeing, lacking the sensitivity and specificity to capture the impact of cosmetic care. Recent developments, such as the BeautyQoL questionnaire [25, 26], have attempted to characterize the relationship between cosmetic products and wellbeing. The BeautyQoL 42‐question scale assesses five dimensions of QoL (social life, self‐confidence, mood, energy and attractiveness) and has been validated in 16 languages. However, there are concerns that the QoL criteria are too broad, with items in the scale referring to an individual's ability to stay awake, family life and trustworthiness. Consequently, changes in overall scores may not be directly attributed to a specific cosmetic product. Moreover, the phrasing of the BeautyQoL specifically addresses improvements to wellbeing resulting from improvements in hair and scalp health, making it insensitive to the negative consequences of poor hair and scalp health, and incapable of capturing the reduction of negative consequences when consumers use new products. Therefore, further improvements are necessary to establish a robust causal link between scalp and hair health and psychological wellbeing.

The objective of this study was to develop and validate a new consumer‐relevant wellbeing instrument, the Hair & Scalp CARE, to assess the direct psychological impacts of dermatological wellbeing in diverse populations. To do this, we administered a large‐scale online consumer survey across the USA to capture a cross‐section of self‐reported needs and concerns. Additionally, we hypothesised that hair and scalp‐related wellbeing would correlate with sleep and stress levels, where good hair and scalp wellbeing would be associated with good sleep and lower stress levels. To investigate this, we utilized the Sleep Health Index (SHI) and Perceived Stress Scale (PSS) alongside the Hair & Scalp CARE.

## METHODS

The experimental procedures were in accordance with the privacy guidelines under the California Consumer Privacy Act of 2018 (CCPA). The study protocol and materials were determined exempt from review by Advarra IRB (Columbia, MD; 2021). All participants signed informed consent before starting data collection. All questionnaires reported in the current paper were administered as part of a broader study conducted to understand personal care needs of healthy populations in the USA. Only relevant measures are reported here.

### Development of the Hair & Scalp CARE (condition and affective response evaluations) questionnaire

The development of Hair & Scalp CARE items involved a multistep iterative process. Initial key themes and known issues surrounding hair and scalp wellbeing were generated through discussions with hair and skin care technical experts in Unilever, and by reviewing previous literature [26, 30, 33]. Insights obtained from these sources informed online semi‐structured qualitative interviews conducted to establish potentially relevant categories. Interviews were conducted with 25 participants who expressed high levels of self‐reported concern regarding various scalp conditions, in partnership with the DiscussIO platform, and were analysed utilizing Facet Theory [38, 39, 40, 41]. The resulting facets derived from the analysis were then integrated into the discussion of item names.

As a result of this process, a scale comprising 23 Hair & Scalp CARE items was created, which were then assessed for face and content validity [42]. Items included in the final scale alluded to hair and scalp issues and needs, the associated feelings and how these needs influenced the participants' behaviour in their daily lives. While most items focused on negative experiences (higher scores indicating lower wellbeing), two positive items were also included (refer to the Table A1 in the Appendix A for the complete list of items).

### Participants

The study aimed to recruit a total of 1200 participants from across the USA, with an equal split of ethnicities between self‐identifying African American, Hispanic and Caucasian consumers. The final sample consisted of 1184 participants, with a small number of respondents being excluded for not ticking all the boxes on the consent form. Participant ages ranged between 18 and 65 for women (*N* = 886, 74.8% of total cohort), and between 18 and 55 for men (*N* = 298, 25.2%). Table 1 shows the full breakdown of participant demographics.

**TABLE 1:** Participant demographics.

Women	African American	Caucasian	Hispanic
*N* (%)	294 (100)	295 (100)	297 (100)
Age			
18–29	97 (33)	65 (22)	48 (16)
30–39	128 (44)	101 (34)	125 (42)
40–65	69 (23)	129 (44)	124 (42)

Men	African American	Caucasian	Hispanic
*N* (%)	102 (100)	97 (100)	99 (100)
Age			
18–29	43 (42)	47 (48)	34 (34)
30–39	47 (46)	38 (39)	49 (49)
40–55	12 (12)	12 (12)	16 (16)

Participants were recruited from various panellist databases with wide geographic representation between November 2021 and February 2022. Panellists were invited to participate in an online research study aiming at identifying personal care needs and concerns of people with diverse ethnic backgrounds across the USA. Upon full completion of the study, participants received reimbursement of $120 for their time and expenses.

### Inclusion and exclusion criteria

All participants completed a short online screening questionnaire to qualify for study inclusion. Participants needed to self‐identify as African American, Hispanic or Caucasian. This questionnaire was administered as part of a larger study on understanding consumer personal care needs, with parallel investigations into underarm and skin‐related concerns and experiences. To ensure that participants were engaged in these areas of personal care, women were required to have used and purchased for themselves in the last 6 months at least one deodorant product, one hair product and one skin product. Men were required to have used at least one deodorant product and one hair product in the last 6 months. In all cases, inclusion was not contingent on the specific type of product used (e.g. shampoo/conditioner); the purchase and use of any product in each category granted eligibility to take part. Applicants who reported to work in advertising, journalism, public relations, marketing research or the personal care/beauty products industry were excluded from participation.

### Other self‐report measures

#### Hair and scalp conditions

In addition to Hair & Scalp CARE, participants also completed a questionnaire recording any hair and/or scalp conditions they were currently experiencing from a pre‐defined list of 15 items. Selected conditions were defined based on the incidence rate of a range of scalp conditions measured in past Unilever consumer studies.

#### Sleep health index (SHI)

The SHI is a 14‐item scale, which assesses sleep quality, sleep duration and disordered sleep. It has been shown to be a valid and reliable tool with Cronbach α of 0.76 [43]. SHI scores range from 0 to 100, with higher scores indicating better sleep health.

#### Perceived stress scale (PSS)

The PSS asks participants to self‐report their perceived levels of stress for the previous month. The original scale was developed to contain 14 items, with the PSS‐10 later developed after exclusion of four items with the lowest factor loadings [44], and a briefer web‐based version (PSS‐4) being subsequently developed [45]. Scores on the PSS scales range from 0 to 40 with higher scores indicative of greater levels of perceived stress. In the present study, participants completed the PSS‐10, which has been shown to have superior psychometric properties compared with the PSS‐4 and PSS‐14 [46]. The PSS‐10 is best described by a 2‐factor structure with perceived helplessness and lack of self‐efficacy subscales [47].

### Procedure

Participants received a link to access the online questionnaire, and after reading the general information and instructions, participants provided consent to participate. The hair and scalp segment of the questionnaire was structured into sections as follows: Section 1 was the screener that determined eligibility; ineligible participants were thanked and exited the survey. Section 2 included the PSS and SHI. Section 3 included questions relating to participants' self‐classification of hair texture/shape, hair and scalp conditions, needs and wants, and the 23 Hair & Scalp CARE items. Section 4 asked for details about hair and scalp products, tools, accessories and procedures. Section 5 asked about demographics and included a question on the device used to complete the questionnaire. The order the sections were administered was fixed, but questions within sections were randomized to prevent order effects. The survey took 60–90 min to complete, with an option to pause and resume within 24 h in case of fatigue. The survey was constructed such that participants could not skip questions without entering a response. Participants were thanked upon completion.

### Analyses

A 2‐stage factor analysis approach was adopted to examine the factor structure of Hair & Scalp CARE. The data were randomly split into two equal samples (*N* = 592). Sample 1 was analysed using an initial exploratory factor analysis (EFA), and Sample 2 was analysed using confirmatory factor analysis (CFA) to assess the validity of the factor structure identified through EFA. The Kaiser–Meyer–Olkin (KMO) measure was used to assess the sampling adequacy of Hair & Scalp CARE, whereby values between 0.5 and 0.7 are considered acceptable and values >0.7 are considered good to excellent [48]. Bartlett's test of sphericity was performed to ensure correlations between items were adequate for EFA.

#### EFA—Sample 1

EFA was conducted using the R Psych package [49]. The number of factors likely to be present in the data set was assessed using several criteria: Parallel analysis with 100 random resamples was conducted using the 95% quantile as a reference comparison. Additionally, Velicer's MAP, which optimizes based on minimizing the average partial correlation [50], very simple structure (VSS), which compared the original correlations with a simplified model [51] and the Bayesian Information Criterion (BIC), were considered. Tests were carried out using the nfactors and fa.parallel functions within the Psych package.

#### CFA—Sample 2

CFA was carried out using the R lavaan package [52, 53]. The model fit was assessed using the comparative fit index (CFI) and Tucker‐Lewis index (TLI), where values above 0.90 indicate moderate fit and values above 0.95 indicate good fit [54]. For the root mean square error of approximation (RMSEA), a value below 0.06 indicates good model fit, and for the standardized root mean square residual (SRMR), values less than 0.08 are considered a good fit [54].

#### Internal reliability

Internal reliability of Samples 1 and 2 was assessed with McDonald's omega (ω_t_), with a lower bound of ω_t_ = 0.7 considered acceptable [55].

#### Calculating Hair & Scalp CARE wellbeing scores

Hair & Scalp CARE wellbeing scores were computed through a systematic process. Participants were provided with a questionnaire where they could express their responses on a Likert scale ranging from ‘Never’ to ‘All the time’ for each item. Each response category was assigned a numerical value on a scale from 1 to 5, with ‘Never’ receiving a score of 5 and ‘All the time’ receiving a score of 1. This scoring system was designed such that higher numerical values indicated a better perceived hair and scalp wellbeing. Items included in the final scale were summed and reweighted to a 0–100 scale.

#### Relationship with hair and scalp conditions

The impact of the reported incidence of 15 hair and scalp conditions on Hair & Scalp CARE scores was investigated, using *t*‐tests to compare Hair & Scalp CARE scores for those currently experiencing a condition to those not in men and women.

#### Correlational analyses with PSS and SHI

To assess the significance of the relationship between Hair & Scalp CARE scores and the PSS and SHI, natural splines were employed. Natural splines were chosen to model the relationships instead of a traditional regression analysis as they allow for the flexibility to capture localized patterns and account for nonlinearity in the relationship between variables. This approach allows for a more accurate representation of the nonlinear relationships between Hair & Scalp CARE scores and the PSS and SHI, respectively, by adjusting the curve's flexibility in different regions of the data.

## RESULTS

### Participant characteristics

The age distribution across the female cohort differed by ethnicity (*χ* ^2^ (4, *N* = 886) = 42.1, *p* < 0.001), with African American participants being younger and Hispanic participants older. There was no difference in age distribution across ethnicities for men (*χ* ^2^ (4, *N* = 298) = 4.4, *p* = 0.35).

### Factor structure and reliability

Hair & Scalp CARE originally consisted of 23 items (see Table A1 in Appendix A). Two items (12 and 13) with reverse scoring were included primarily to confirm that participants were paying attention while completing the questionnaire. Our analysis showed there were differences in consumer response patterns between the negative and positive items, which could potentially be attributed to how the reversed questions were interpreted. Therefore, items 12 and 13 were removed prior to EFA analysis to limit additional variance [56]. Consequently, EFA was conducted with the 21 remaining items.

### EFA and CFA

The suitability of Sample 1 data to FA was confirmed with a Kaiser–Meyer–Olkin index of 0.98, and a significant result for Bartlett's sphericity test (*p* < 0.001) indicated that correlations between items were sufficient for EFA. All criteria used to assess the number of factors suggested a single factor was appropriate, with loadings on individual questions ranging from 0.65 to 0.80 (See Table 2) and the proportion of explained variation being 55%. The CFA demonstrated an acceptable fit for the Sample 2 data (Comparative Fit Index = 0.961, Tucker‐Lewis Index = 0.956, RMSEA = 0.053, SRMR = 0.035).

**TABLE 2:** Loadings for scalp and hair wellbeing factors.

Item	Factor loading
My scalp and/or hair problems make me feel less confident	0.76
My scalp and/or hair problems make me feel less attractive	0.75
My scalp and/or hair problems make me feel other people judge me	0.79
My scalp and/or hair problems make me feel insecure in social situations	0.80
My scalp and/or hair problems make me feel sad or unhappy	0.80
My scalp and/or hair problems make me feel anxious or concerned	0.79
My scalp and/or hair problems embarrass me when around others	0.80
My scalp and/or hair problems heighten my awareness about how I look	0.71
My scalp and/or hair problems impact my wellbeing	0.66
My scalp and/or hair problems make me feel irritated	0.79
My scalp problems make me feel dirty, unclean or unhygienic	0.76
My scalp and/or hair problems make me reluctant to take selfies or have pictures taken of me	0.76
My scalp and/or hair problems make me wash my hair more often	0.65
I change my social plans and stay at home because of my scalp and/or hair problems	0.75
I cannot wear my hair the way I want because of my scalp and/or hair problems	0.73
I hide my scalp and/or hair problems by wearing a hat or scarf	0.74
The state of my scalp and/or hair influences how I interact with others	0.73
My scalp and/or hair problem makes me use on‐the‐go solutions	0.69
My scalp and/or hair problem makes me check my clothes regularly	0.73
My scalp problem makes me scratch or check my scalp discreetly	0.71
My scalp problem affects how well I sleep	0.70

### Internal reliability estimation

The McDonald's omega hierarchical scores calculated as the internal reliability estimates confirmed a high internal consistency for Sample 1 (ω_t_ = 0.89) and Sample 2 (ω_t_ = 0.90) datasets. ω_t_ values obtained in the present study provide support for the calculation of a total score for a one‐factor scale.

### Hair & Scalp CARE score descriptives

The overall mean Hair & Scalp CARE score for the cohort was 72.01 (± 22.05 SD). Men demonstrated a higher mean Hair & Scalp CARE score (77.62 (± 21.70)) than women (70.12 (± 21.86)), indicating a higher average hair and scalp‐related emotional wellbeing.

### Relationship with hair and scalp conditions

Most participants reported concern for at least one hair or scalp condition (83.4% of women and 65.1% of men). Participants who reported conditions had significantly lower Hair & Scalp CARE scores than those who did not, with an average decrease in mean Hair & Scalp CARE score of 91.18 (± 11.24) to 65.93 (± 21.03) for women (*t*(383.2) = 20.912, *p* < 0.001) and 88.68 (± 15.01) to 71.69 (± 22.43) for men (*t*(281.74) = 7.785, *p* < 0.001). This confirms the generally negative impact of hair and scalp conditions on emotional wellbeing.

**FIGURE 1:**
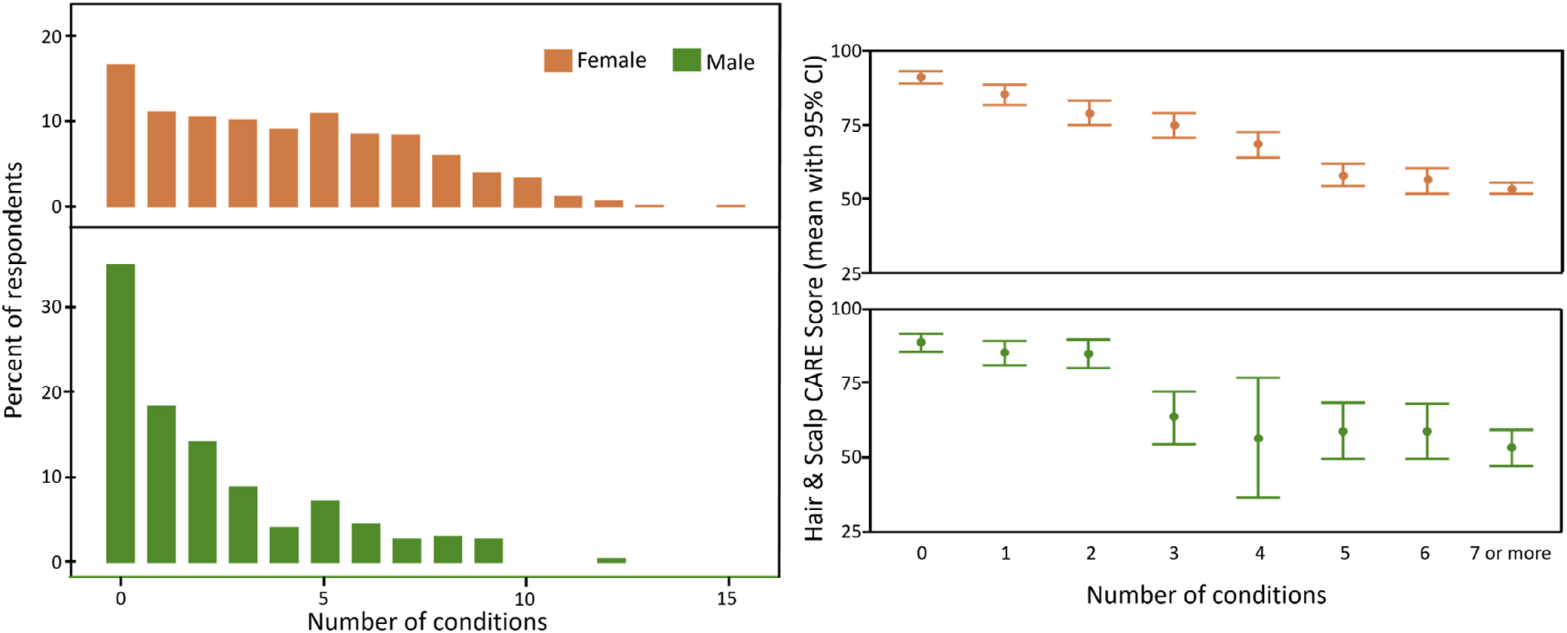
Relationship between Hair & Scalp CARE and the number of currently experienced hair and scalp conditions.

Additionally, a regression analysis indicated that the number of reported conditions explained a significant proportion of variance in Hair & Scalp CARE scores (women: *R* ^2^ = 0.43, *F*(7, 878) = 96.37, *p* < 0.001; men: *R* ^2^ = 0.40, *F*(7, 290) = 27.53, *p* < 0.001). As can be seen in Figure 1, Hair & Scalp CARE scores decreased as the number of reported conditions increased. Regression analysis confirmed the trend for women for each additional condition, while for men, significantly lower wellbeing was observed for three or more conditions.

### Relationship with PSS and SHI

**FIGURE 2:**
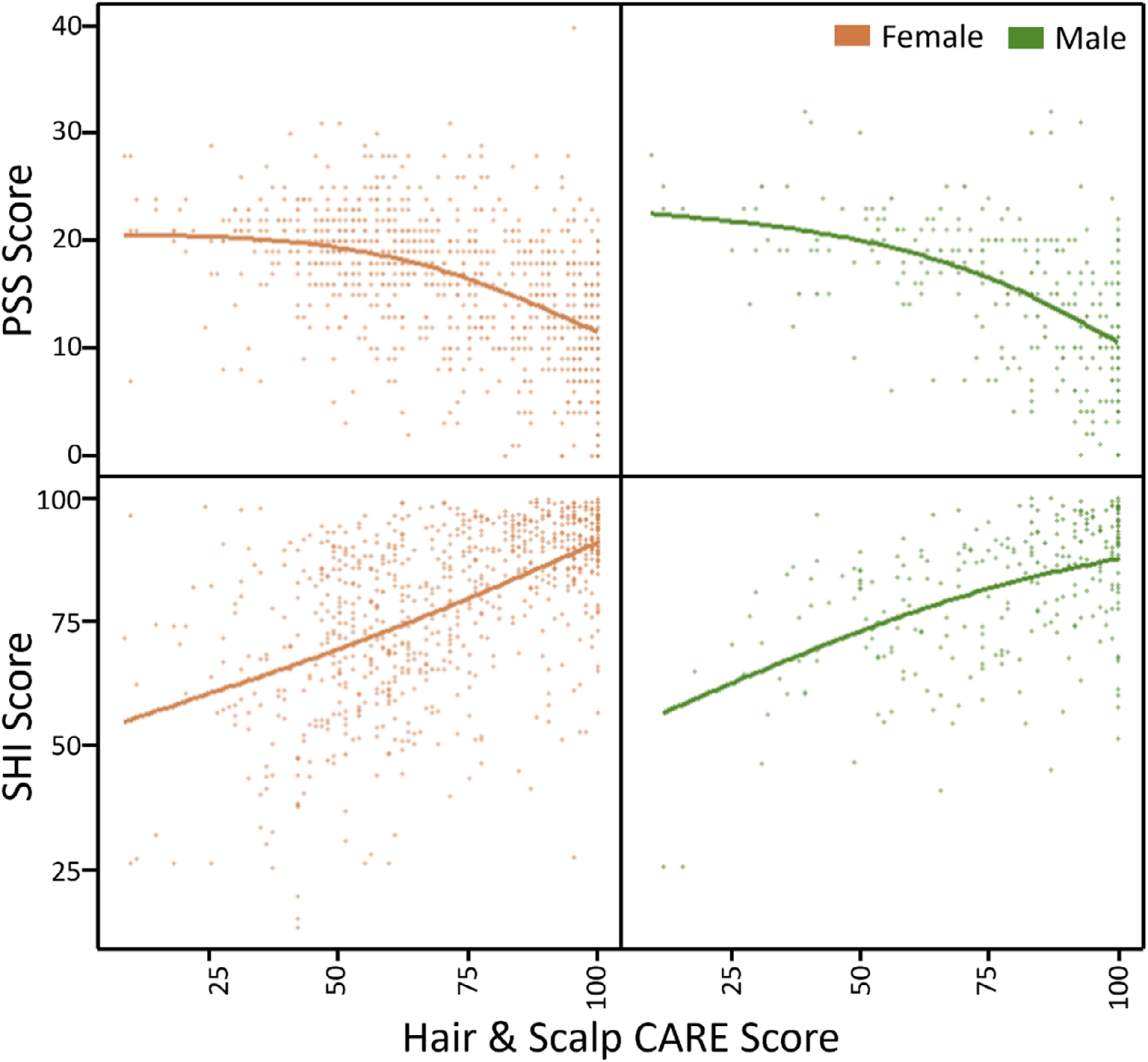
Relationship between Hair & Scalp CARE scores and other self‐report measures.

The relationships between Hair & Scalp CARE scores and scores from two metrics of stress and sleep health, the PSS and SHI, respectively, were investigated. Table 3 shows a breakdown of these respective relationships according to gender. An *F*‐test from the natural spline model with 2df versus intercept only model showed a significant relationship (*p* < 0.001) regardless of gender. Hair & Scalp CARE scores were negatively associated with PSS and positively associated with SHI, indicating higher hair and scalp wellbeing is related to lower levels of stress and better sleep. A graphical representation can be seen in Figure 2. Subsequent testing of the spline fit versus a linear model suggests the linear model is adequate for SHI (Women: *F*(1,804) = 0.78, *p* = 0.38, Men: *F*(1,277) = 1.53, *p* = 0.217), whereas the spline fit is superior for PSS (Women: *F*(1,883) = 19.74, *p* < 0.001, Men: *F*(1,295) = 7.41, *p* <0.01).

**TABLE 3:** *F*‐test results: Hair & Scalp CARE—PSS scores and Hair & Scalp CARE—SHI scores.

Gender	PSS	SHI^a^
Women	*F*(2,883) = 145.79, *p* < 0.001	*F*(2,804) = 163.69, *p* < 0.001
Men	*F*(2,295) = 68.06, *p* < 0.001	*F*(2,277) = 45.05, *p* < 0.001

*Note*: *F*‐test shows that of natural spline model with 2df vs intercept only model.

^a^ 97 ‘Rather not say’ responses for SHI excluded from analysis.

## DISCUSSION

The investigation of scalp‐related wellbeing is an established practice in clinical samples with dermatological conditions, supported by several well‐established clinical and objective instruments [30, 33]. Conversely, the assessment of scalp‐related wellbeing in healthy populations is comparatively sparse, despite the evidence of the importance of scalp and hair health for mental wellbeing and self‐confidence. The aim of the present study was to address this gap through the development and validation of a novel wellbeing scale specifically focused on diverse consumers experiencing a range of non‐clinical scalp conditions. It is our aspiration that this 21‐item scale can be employed effectively in academic research, including studies conducted in commercial settings, to assess the impact of commercially available hair and scalp cosmetic products on consumer wellbeing, in a manner that captures cultural variability in experiences.

### Summary of findings

The study utilized exploratory factor analysis followed by confirmatory factor analysis, which supported the existence of a single factor: scalp and hair problems. Hair & Scalp CARE items addressed various aspects of wellbeing, including physical impairment (e.g. ‘My scalp problem affects how well I sleep’), interpersonal relationships (e.g. ‘My scalp and/or hair problems make me feel other people judge me’) and internal emotions (e.g. ‘My scalp and/or hair problems make me feel sad or unhappy’). Therefore, Hair & Scalp CARE is equipped to provide a comprehensive understanding about consumer experiences beyond that of generic QoL and disease‐specific instruments.

Crucially, the ability of Hair & Scalp CARE to capture the above dimensions in a single factor also contributes to its practicality as a streamlined and easy‐to‐administer tool in future research. Furthermore, the negative phrasing of the 21 items in Hair & Scalp CARE effectively captures the issues that most acutely impact consumers' mental wellbeing. This distinguishes Hair & Scalp CARE from scales that are positively framed to assess increases in consumer satisfaction, such as the Beauty QoL questionnaire [25, 26]. The significant relationship between Hair & Scalp CARE scores and the self‐reported incidence of hair and scalp conditions provides further evidence of the association between physiological issues affecting the hair and scalp and an individual's emotional wellbeing. This finding confirms the negative impact of hair and scalp conditions on one's psychological state.

Finally, consistent with the study's hypotheses, individual scores on Hair & Scalp CARE positively correlated with the SHI and negatively correlated with the PSS while showing good convergence validity. This trend was observed across both genders, highlighting a universal link between stress levels, sleep quality and wellbeing that extends to consumer cosmetic contexts.

### Limitations and future directions

While the study achieved satisfactory internal consistency, further investigation and validation of Hair & Scalp CARE are recommended. Recruitment prioritized a large and diverse sample within the USA, allowing for analyses across three major demographics and random data splitting in order to conduct both EFA and CFA analyses. However, future research should involve administration and replication in different countries and languages to ensure international validation and cross‐cultural applicability.

It is also important to note that the hair and scalp conditions discussed in this study were based on self‐report measures. This was chosen as the focus of the study was on the subjective perception of one's own experiences, and so, it was pertinent to capture how individuals view themselves. However, incorporating objective methods of measurement alongside self‐reporting would provide valuable insights by comparing subjective perceptions with the objective frequency and severity of different hair and scalp issues across demographics. In addition, the capturing of product regime influence on both self‐reported wellbeing and objective measures of scalp and hair health would be a valuable future implementation of the current work.

## CONCLUSION

Overall, the current findings significantly advance the ability to accurately capture and assess the impact of hair and scalp wellbeing on psychological wellbeing in non‐clinical populations. The development of the Hair & Scalp CARE tool offers a convenient and valid metric to evaluate the psychological consequences of hair and scalp problems, and how hair and scalp‐related wellbeing can be interlinked with perceived stress and sleep quality. It is our hope that this instrument will aid future research to better understand consumer experiences, and the potential impact of consumer products to alleviate symptoms and directly improve self‐confidence. Utilization of Hair & Scalp CARE will also provide valuable insights into the development and refinement of products that will effectively promote wellbeing across diverse populations.

## AUTHOR CONTRIBUTIONS

William M. Hirst, Therese Jones, Margaret Scott, Monique A. M. Smeets, Jeremy Shen, Anna Thomas and Timo Giesbrecht contributed to the development of the experimental design and planning of this work. Therese Jones and Margaret Scott designed and analysed the qualitative interviews, and developed the Hair & Scalp CARE questionnaire items. Jeremy Shen, Therese Jones and Monique A. M. Smeets oversaw all of the data acquisition. William M. Hirst conducted statistical analyses. Alice Newton‐Fenner and William M. Hirst produced all figures. Alice Newton‐Fenner produced the final written manuscript, which was overseen by Timo Giesbrecht and Anna Thomas. All authors reviewed and provided feedback on the final manuscript.

## FUNDING INFORMATION

The work was funded by Unilever U.K. Central Resources Limited.

## CONFLICT OF INTEREST STATEMENT

AN‐F, WMH, TJ, MS, MAMS, JS, AT and TG work for Unilever.

## APPENDIX A

**TABLE A1:** Hair & Scalp CARE questionnaire.

How often during the past 4 weeks do these statements describe you?	Never	Rarely	Sometimes	Often	All the time
My scalp and/or hair problems make me feel less confident					
My scalp and/or hair problems make me feel less attractive					
My scalp and/or hair problems make me feel other people judge me					
My scalp and/or hair problems make me feel insecure in social situations					
My scalp and/or hair problems make me feel sad or unhappy					
My scalp and/or hair problems make me feel anxious or concerned					
My scalp and/or hair problems embarrass me when around others					
My scalp and/or hair problems heighten my awareness about how I look					
My scalp and/or hair problems impact my wellbeing					
My scalp and/or hair problems make me feel irritated					
My scalp problems make me feel dirty, unclean or unhygienic					
*My hair makes me feel good about myself*					
*My hair makes me feel attractive*					
My scalp and/or hair problems make me reluctant to take selfies or have pictures taken of me					
My scalp and/or hair problems make me wash my hair more often					
I change my social plans and stay at home because of my scalp and/or hair problems					
I cannot wear my hair the way I want because of my scalp and/or hair problems					
I hide my scalp and/or hair problems by wearing a hat or scarf					
The state of my scalp and/or hair influences how I interact with others					
My scalp and/or hair problem makes me use on‐the‐go solutions					
My scalp and/or hair problem makes me check my clothes regularly					
My scalp problem makes me scratch or check my scalp discreetly					
My scalp problem affects how well I sleep					

*Note*: These questions are about the skin issues that have bothered you over the past 4 weeks, how they made you feel and how they impacted your behaviour in everyday life. Items in grey italics indicate positively phrased statements removed prior to statistical analysis.
